# On the Performance of a Horizontally Mounted ADCP in an Energetic Tidal Environment for Floating Tidal Turbine Applications

**DOI:** 10.3390/s24144462

**Published:** 2024-07-10

**Authors:** Jan Dillenburger-Keenan, Calum Miller, Brian Sellar

**Affiliations:** 1 Orbital Marine Power, Kirkwall, Orkney KW15 1ZL, UK; 2 School of Engineering, The University of Edinburgh, Edinburgh EH9 3BF, UK

**Keywords:** ADCP range, floating tidal turbine, velocimetry, power performance assessment

## Abstract

Incident flow measurement is key in the tidal industry for conducting power performance assessments. This paper explores the use of a horizontally mounted Nortek Signature 500 Acoustic Doppler Current Profiler (ADCP) as a means for incident flow measurement onboard a utility-scale tidal turbine. This study shows that the measurement range of an ADCP mounted horizontally in highly dynamic tidal flow (up to 4 m/s) is less than the maximum range stated by the manufacturer. The ability for the horizontal ADCP to accurately resolve velocities in a multi-beam configuration is also analysed. Effects from both vertical shear and beam selection result in incident flow velocities that differ from a single horizontal beam recording. The maximum measurement range of the instrument is found to depend on current speed and on the proportion of data loss that is acceptable to the user. The ability of the ADCP to record data from the free-stream velocity two equivalent diameters upstream of the O2, as set out by IEC TS 62600-200, is considered. It is found that at this distance, there is 90% data loss. Accepting only 10% data loss across all flow speeds resulted in a maximum range of 31 m for a Nortek Signature 500 in this study. While some limitations of an ADCP deployed horizontally in highly energetic tidal flow are identified, the benefits of mounting the sensor close to the rotor facing horizontally into the incoming flow mean that valuable data are still produced for tidal turbine operators.

## 1. Introduction

Tidal energy is an untapped renewable energy resource, one that is prevalent throughout the United Kingdom (UK) coastal waters. It is estimated that up to 11% of the UK electricity needs could be met by tidal energy, enough to displace a significant proportion of heavy fuel electricity generation within the UK [1]. Barriers to energy market entrance for tidal energy developers have seen a reprieve in recent years with government ring-fenced subsidies enabling early phase designs to avoid competing with established technologies [2,3]. In conjunction with market support, the tidal energy industry continues to innovate in order to bring costs down.

Tidal turbines have seen a division in design philosophy with the majority of globally deployed turbines opting for either a seabed-mounted or floating superstructure. Floating tidal turbines ensure that operations and maintenance costs are low as major components are accessible using small vessels. Placing sensors on a floating tidal turbine benefits from accessibility and the ability to intervene quickly to replace damaged or broken units. However, certain parameters are more difficult to record from a floating platform.

Incident flow velocity is a key parameter for a tidal turbine from both a control and performance perspective. The dominant sensor used in full-scale tidal environment velocimetry is the Acoustic Doppler Current Profiler (ADCP). ADCPs use multiple (typically 3–5) acoustic transducers whose acoustic *beams* diverge away from the instrument. The Doppler shift in acoustic signal returning to the transducer gives the speed of flow along the axis of the transducer. Given the diverging multi-beam configuration of the sensors, the x, y, z components of flow relative to earth or sensor coordinates can be resolved [4,5]. This non-intrusive measuring technique allows for the flow speed to be calculated at defined distance-bins away from the sensor, enabling a ’profile’ to be recorded.

Standard practice for the deployment of ADCPs in the tidal environment is to place them on the sea bed profiling the vertical water column [6,7,8,9]. Isolated ADCPs rely on battery power and, therefore, have a defined deployment period, after which they require collection for data extraction. Placing an ADCP onboard a tidal turbine, however, enables continuous sensor power and, therefore, a continuous measurement campaign. A number of studies have seen the deployment of ADCPs onboard a tidal turbine [10,11].

In order to record incident flow from onboard a tidal turbine, the ADCP must be placed horizontally profiling into the flow. The International Electrotechnical Commission (IEC) stipulates that true incident flow data must be obtained from beyond the influence of the turbine on the flow [12]. Turbine-mounted ADCPs for incident flow measurement are inhibited by an inability to be situated beyond the influence of the turbine on the flow. As a result, the ADCP must accommodate this requirement with an `adequate profiling range’. The deployment of a horizontal ADCP has been suggested as an addition to the next iteration of the IEC guidance on incident resource measurement for power performance assessment [13].

Previous horizontal ADCP deployments on tidal turbines for inflow analysis include the DeepGen-IV [10], the Sabella D10 [11], and the DeltaStream 400 kW turbine [8]. The DeepGen-IV saw a horizontal single-beam Nortek Continental profiling behind the rotor, and a single-beam Nortek AD2CP placed on the rotating hub profiling forward of the rotor. The Sabella D10 saw the deployment of a 5-beam Nortek Signature 500 on the aft of the turbine profiling rearward: due to the fixed nature of the turbine, this constituted incident profiling on the ebb tide. The DeltaStream turbine saw a single-beam 1 MHz Nortek Aquadopp sensor placed in the rotating nose cone of the turbine profiling into the incident flow.

The deployment of ADCPs from onboard a tidal turbine enables a further reduction of seabed operations for a floating tidal turbine. To the authors’ knowledge, the deployment of a horizontal ADCP at hub height on a floating, utility-scale, tidal turbine remains a research gap. While theoretical advantages include continuous sensor power and data collection, the envelope of application has not been tested. This study quantifies the range of a horizontal ADCP placed at hub height on a floating tidal turbine in relation to sensor manufacturer stated range, while also exploring advantages and shortcomings of sensor configurations.

## 2. Materials and Methods

This study outlines the operational envelope of a horizontally mounted 5-beam ADCP when placed at hub height on a utility-scale floating tidal turbine. Firstly, the study identifies the range of a single horizontal beam under a number of incident flow speeds in order to inform technology developers of expected range for the instrument used. Secondly, an assessment of horizontal flow measurement accuracy and range from divergent beams of a horizontally mounted ADCP is presented. The results are then discussed and conclusions are drawn.

### 2.1. The Floating Tidal Turbine

**Figure 1:**
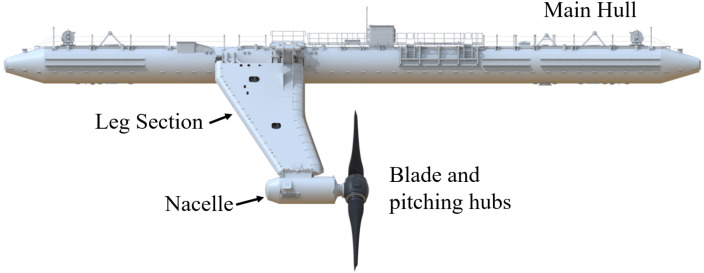
Side profile of the O2 2 MW tidal turbine designed, built, and installed by Orbital Marine Power.

Orbital Marine Power is a long-standing sector leader in floating tidal energy. The third device deployed by Orbital is the O2. The O2 is a 2 MW tidal turbine composed of two separate 1 MW nacelles supported by legs attaching them to the main hull floating structure (see Figure 1). The two rotors have a diameter of 20 m each. The rotors are designed to be raised to above the water line for maintenance, and are lowered for power production, giving a clearance of 3 m between top-dead-center of the rotor and the sea surface during operation.

The O2 is kept on station using a catenary mooring system. Four mooring lines extend from the turbine to the sea floor, two from each end of the main hull. Given the catenary nature of the mooring system, the turbine can move ≈25 m in any direction on the plane of the sea surface. The location of the turbine is dependent on wind direction, wave climate, and thrust imparted on the structure and rotors by the tide; however, the turbine does not vane between tides.

### 2.2. The ADCP

**Figure 2:**
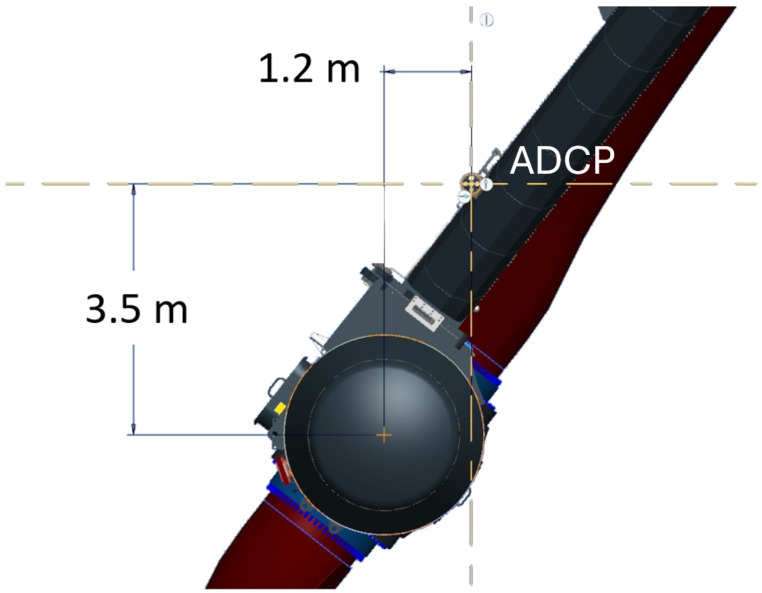
Leg ADCP location as viewed from aft of the O2 with distances to the hub center shown.

**Figure 3:**
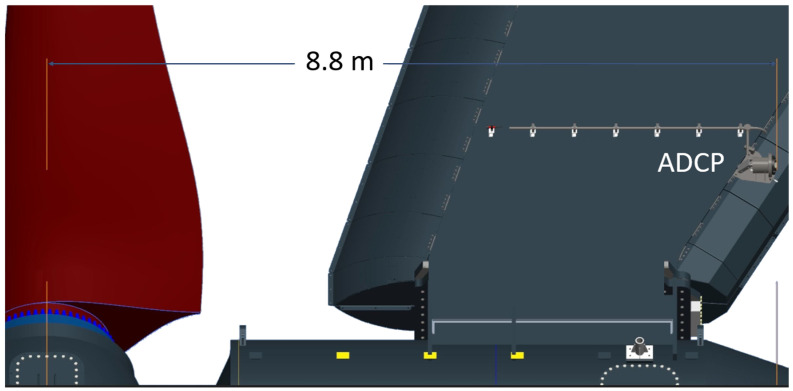
Leg ADCP location as viewed from the port side of the O2 with distance to the hub center shown.

A 500 kHz Nortek Signature 500 5-beam ADCP was placed on the port leg of the O2. The instrument was placed near the rotor centre, however, was offset as seen in Figure 2 and Figure 3. The instrument was positioned horizontally pointing aft using a mounting bracket. The leg position of the ADCP avoids rotation that would be seen from a hub mounting.

The 5-beam ADCP was orientated with the central beam pointing horizontally aft with the other two pairs of diverging beams placed along the vertical and horizontal plane. This resulted in a ‘+’ shape when viewed from in front of the sensor head. All five of the transducers were activated, recording continuously at 4 Hz for the duration of the data set.

**Figure 4:**
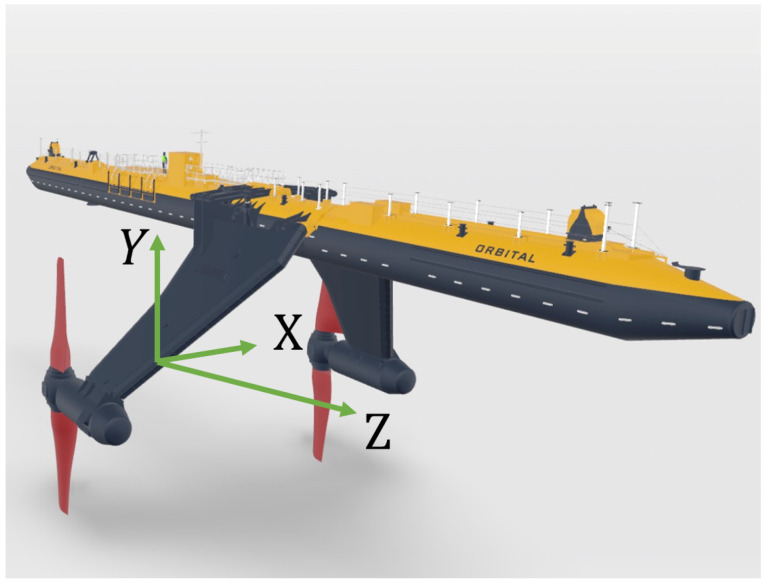
Leg ADCP coordinate system.

The coordinate system used in the analysis of horizontal ADCP data remained that of the instrument itself (see Figure 4). This results in the flow towards the sensors being on the *z*-axis, i.e., the turbine inline flow.

### 2.3. ADCP Data Set

Data were recorded using the horizontal ADCP from 1 March 2023 until 14 March 2023 representing seven tidal cycles. The horizontal ADCP logged 5 beams continuously in 1 m bins for 68 bins at 4 Hz. This time period represents a 14-day data set and covers the continuous logging of close to a full springs tidal cycle. Only flow towards the sensor was considered for this study as this represents incident resource. Flow away from the sensor was contaminated by the turbine rotor and instrument setup wake and so was not included in this study.

**Figure 5:**
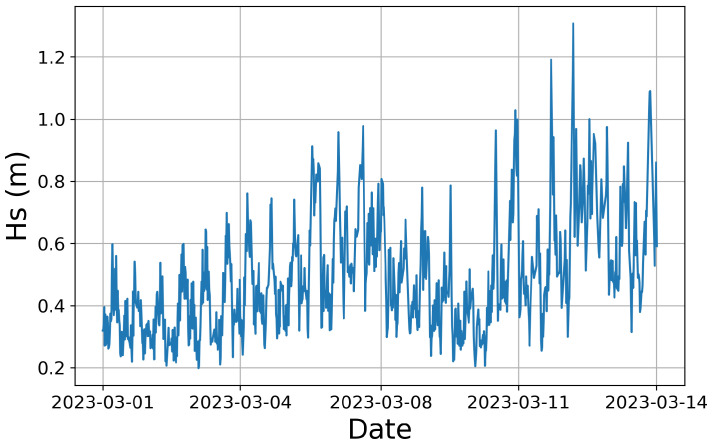
Significant wave height for the duration of the data set.

Figure 5 shows the environmental conditions during the data acquisition campaign. The wave height was recorded using Orbital Marine Power’s in-house wave recording technique. It can be seen that the wave height remained below Hs = 1.3 m for the duration of the data acquisition period.

### 2.4. Data Quality Control Procedure

Quality Assurance/Quality Control of Real-Time Oceanographic Data (QARTOD) is a framework of quality control manuals for a range of oceanic measurement variables. Within QARTOD exists a framework for quality control for in situ current measurement using an ADCP [14]. Quality control of ADCP data is used to ensure erroneous data are not included in processes performed post data collection.

The QARTOD process begins with the raw collection of data using an ADCP. Each datum in the recording is then evaluated with respect to tests and flagged as either passed, not evaluated, suspect, failed, or missing. Suspect data points are then investigated to determine their validity. Nonconformists within the data set to the QARTOD tests are removed or assigned a NaN value.

The data from the horizontal ADCP were filtered using a number of QARTOD filters (see Table 1 and Table 2). Only points that passed all filters were used in the Section 3 of this study.

#### 2.4.1. Beam Quality Control—Application of QARTOD

Beam data recorded by the horizontal ADCP were filtered using QARTOD tests with constraints outlined in Table 1 with the addition of a bespoke filter for horizontal ADCP deployment. Filters were applied to each bin and each beam for the duration of the data set. Failure to meet filter criteria resulted in a flagged data point.

Filter JDK1 was applied only to the central beam of the horizontal ADCP to remove spikes from the data set. Under laminar flow conditions with streamlines parallel to a horizontal seabed, it is expected that the horizontal beam would record the same velocity at each bin distance from the sensor. This is because there are no shear profiles across the study domain as would be the case for an upward-facing, seabed-mounted ADCP. These conditions are not realised in the field; however, spikes away from the median value along the beam are considered suspect and are flagged by this filter.

Demonstration of successful data collection with stringent filters can act as a clear baseline of quality control for future studies.

#### 2.4.2. Coordinate Transform

To convert beam velocity measurements to instrument coordinates, the beams were coordinate transformed. The output of this stage was instrument coordinate velocities from the sensor to the end of the measurement domain. The physical orientation of the beams used in the coordinate transformation determines the plane being recorded. Each opposite pair of divergent transducers can record in one plane while Beam 5 can only record directly in front of the ADCP.

One of the underpinning assumptions in the transformation of beam velocities to instrument coordinates is homogeneous flow at each bin distance from the sensor [4]. It is assumed that every beam measures the same velocity at each bin distance away from the sensor head.

#### 2.4.3. Transformed Quality Control

The coordinate transformed flow speeds were further quality controlled with a second level of QARTOD filters (see Table 2). Each of these filters (T10 and T20) was used to de-spike the resolved data. Including a limit to velocity maximum and bin by bin velocity difference ensured that errors introduced by the transformation process were removed.

### 2.5. Single-Beam Range

The maximum range achievable by a horizontal ADCP in an energetic tidal environment is an important variable as it sets the domain limit for future studies with the sensor. This includes any studies using a horizontal turbine-mounted ADCP for a power performance assessment to measure the free stream tidal velocity. Developing an understanding of the ability for a horizontal ADCP to range to the free stream is important in this context.

The maximum distance to which data can be recorded is defined, in this case, as the quantity of data remaining post quality control filters. The aim of this study was not to define a maximum range but rather to demonstrate the effect of range on the volume of data remaining post quality control. It is then for the ADCP user to determine a suitable level of data rejection for their study and, therefore, the range they can expect.

### 2.6. Incident Flow from Divergent Beams

For a horizontal ADCP, the *z*-axis represents the inline flow to the turbine (see Figure 4). To record velocities along the *z*-axis using a 4-beam ADCP, a number of beams are combined as there is no beam directly in line with the *z*-axis and, therefore, no direct measurement [4]. Traditional 4-beam ADCP deployments use opposite beams to record two separate values for the *z*-axis velocity. The difference in these two values of *z*-axis velocity is then used as a quality control parameter often called the `error velocity’. The issue with this method is that these derived values for *z*-axis velocity could be subject to the same error disguising them from a comparison filter.

Beam 5 of a 5-beam ADCP, like that used in this study, represents a direct measurement of the *z*-axis velocity (see Figure 4). As such, this study compares the direct measurement of the *z*-axis velocity (Beam 5) to the *z*-axis velocities from transformed divergent beams in a bid to quantify the true error in *z*-axis velocity from transformed beams when the ADCP is placed horizontally.

**Figure 6:**
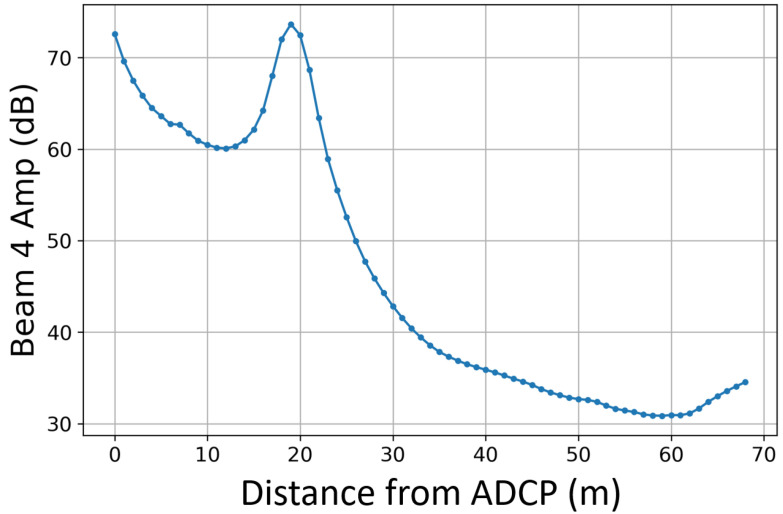
Mean amplitude of Beam 4 against distance in front of the horizontal ADCP showing sea surface strike around 17 m.

Due to the deployment characteristics as well as the orientation of the horizontal ADCP, Beam 4 is located closest to a boundary (the sea surface). As a result, the range of this beam is limited by surface strike. Given the depth of deployment as well as the angle of the beam to the horizontal, this beam can only reach 17 m. This can be see in Figure 6, where the amplitude of Beam 4 increases as it strikes the sea surface. For this study, any data recorded by Beam 4 after 17 m from the sensor head were omitted.

## 3. Results

### 3.1. Single-Beam Range

**Figure 7:**
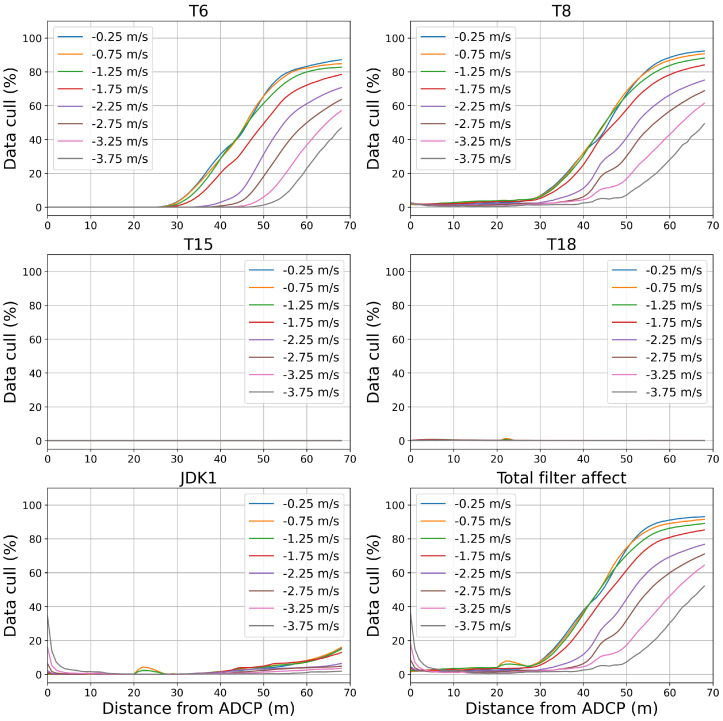
Percentage of data removed in Beam 5 after filters applied, as a function of distance in front of the horizontal ADCP for a range of flow speed brackets. Each sub-figure represents a different filter with each line representing a 0.5 m/s speed bracket with the bracket centre indicated. The total filter effect is shown in the bottom right sub-figure.

The data from Beam 5 were quality controlled using the filters outlined in Section 2.4.1. The percentage of data removed after the application of each filter can be seen in Figure 7 with negative flow speeds representing flow approaching the sensor. Positive flow speeds recorded were omitted in this study as discussed earlier.

Figure 7 shows that most of the data were able to pass through the amplitude filter (T6) in the initial 40 m of the study area. The T6 filter shows a sharp increase in data removed from 40 m to the end of the domain. This increase is seen most dramatically in the lower absolute flow speeds with less than 20% of data remaining when tide speed is between −1.5 m/s and 0 m/s beyond 50 m from the ADCP. The correlation based filter represented by T8 in Figure 7 shows a similar high pass rate for the initial study area. The decline in passing data for T8 begins at 30 m and accelerates at 40 m. Again, it can be seen that the filter removes more data at lower flow speeds. Filter T15 removes data as a result of high rates of pitch, roll, or yaw. Figure 7 shows that no data reached this threshold and so this filter had no effect on the data set. Filter T18 was designed as a method to remove spikes in amplitude due to suspended bodies in the water column. Figure 7 shows that very little data were removed by this filter. Filter JDK1 removed a large proportion of data from higher flow speeds in close proximity to the horizontal ADCP, as can be seen in Figure 7. JDK1 had a further small effect throughout the study domain with a noticeable removal of data from the mid-range region of around 25 m but a small amount when compared to T6 and T8.

Figure 7 shows the combined result of all filters used for the quality control of the beam data in this study. It is clear that between 5 m and 31 m, over 90% of data meet the requirements set out by the QARTOD filters for all flow speeds. Beyond 31 m, −2.5 m/s to −4.5 m/s sees a large removal of data. Lower speeds see a larger volume of data removal, over 90% removed, while higher absolute speeds see only around 60% of data removed by the end of the study domain.

Table 3 shows the range from the horizontal ADCP for a given acceptable data loss and flow speed. The value for acceptable data loss is to the discretion of the user and will have an impact on the uncertainty of the flow measurements. It can be seen that the full range of the sensor configuration (68 m) is reached if 60% data loss is accepted at −3.75 m/s. It can also be seen, as is shown in Figure 7, that slower flows inhibit the range of the horizontal ADCP.

### 3.2. Incident Flow from Divergent Beams

*z*-axis velocities recorded by Beam 5 were compared to a number of *z*-axis velocities derived from diverging beams. For simplicity of notation, *z*-axis velocities from Beam 5 will be written as $Z_{5}$, while *z*-axis velocities from diverging beams will have the beam numbers used as the subscript (e.g., *z*-axis velocity derived from Beams 1, 2, 3, and 4 will be written as $Z_{1,2,3,4}$).

**Figure 8:**
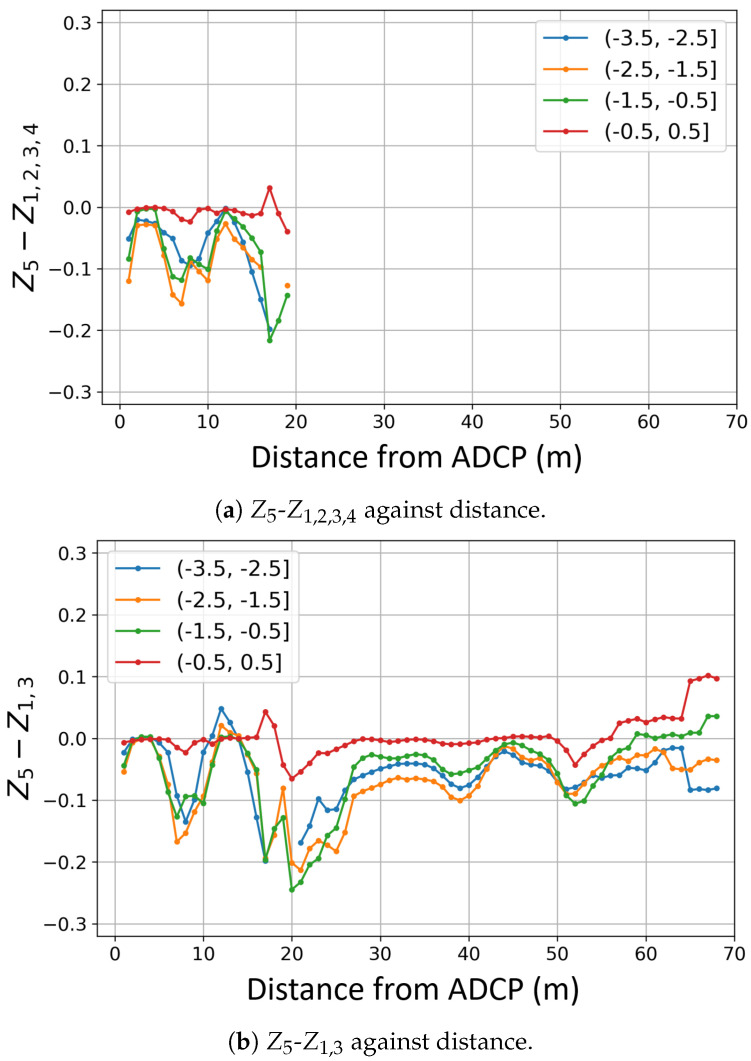
Z velocity from beam combinations compared to Beam 5 against distance from the horizontal ADCP binned by flow speed.

Two different measurements of *z*-axis velocity were made using combinations of diverging beams: $Z_{1,3}$ and $Z_{1,2,3,4}$. The flow speed bin averages are displayed in Figure 8. Figure 8 represents the average velocity error compared to $Z_{5}$ for each distance bin. Quality control filters were applied as per Table 1 and Table 2. Distance bins where more than 70% of data were removed by filters were considered beyond the range of the horizontal ADCP for this study.

Figure 8a shows the difference between $Z_{5}$ and $Z_{1,2,3,4}$. The absolute difference in velocity never goes beyond 0.1 m/s at low flow speeds, while higher flow speeds are responsible for more variation with distance as well as greater errors. This figure only extends to 17 m as Beam 4 strikes the sea surface after this distance, as discussed in Section 2.6.

Figure 8b shows the error in recording for $Z_{1,3}$. Using only Beams 1 and 3, $Z_{1,3}$ is able to record velocities to 68 m for slower flow speeds as well as higher speeds. The absolute error in measurement across all tide speeds is only momentarily greater than 0.2 m/s.

The majority of the span shown in the sub-figures of Figure 8 sees a negative value of *Z* velocity error. This suggests that the *Z* velocity recorded using beam transformations is higher than *Z* velocities recorded by Beam 5 directly, irrespective of the choice of beams.

## 4. Discussion

The Nortek Signature 500 data sheet indicates a maximum range of 70 m in average mode [15]. This study demonstrated that a horizontally mounted Nortek Signature 500 ADCP is not capable of profiling to the maximum range indicated by the manufacturer under every flow condition due to the high-energy nature of the environment. The range was, in fact, shown to be flow-speed-dependent for a Nortek Signature 500. Despite this dependency, 50 m represents the distance at which the horizontal ADCP could measure while maintaining over 50% of all data post rigorous quality control for all speeds for this study setup. The purpose of this study was to demonstrate a `percentage data rejection—range’ dependency for a single instrument, and so the percentage data rejection that correlates with `end of range’ is not explicitly identified. Rather, it is for the operator to determine the percentage rejection that is deemed appropriate given sensitivity and uncertainty requirements for a study on a case-by-case basis.

It is important to note that the choice of sensor frequency and power are also deciding factors in the range of an ADCP. Previous horizontal deployments of 1 MHz single-beam sensors have seen ranges much lower than this study. The 1 MHz Nortek Aquadopp single-beam ADCP placed in the nose cone of a seabed-mounted tidal turbine in [8] ranged 20.4 m, while a Nortek AD2CP 1 MHz single-beam sensor in [9] ranged 13 m upstream.

### 4.1. Horizontal ADCP for Power Performance Assessment

A power performance assessment is one major use-case of incident flow measurement with IEC Technical Specification (TS) 62600-200 being a major guidance document in the creation of a turbine power curve [12]. The document, in its current form, mandates incident flow measurement from a vertically profiling ADCP placed in the free stream flow. The document is very specific in the identification of incident-free stream measurement locations. For the recording of incident flow from in front of the turbine, an ADCP must be placed two to five rotor diameters upstream of, and inline with, the rotor [12]. The power performance assessment also requires flow measurement over the entire rotor capture area of the turbine.

#### 4.1.1. Single-Beam Range

Given the required non-dimensional upstream distance for free stream flow recording according to TS 62600-200 [12], the range requirement for the use of a horizontal ADCP for incident flow recording is turbine specific. That said, the ADCP range found in [8] using a 1 MHz Nortek Aquadopp and in [9] using a 1 MHz Nortek AD2CP represented 1.75D and 0.72D of their respective turbines, where D is the turbine rotor diameter. This is much shorter than the TS 62600-200 prescribed distance of 2-5D.

The acoustic frequency of an ADCP has a bearing on the manufacturer stated range. There is, therefore, an effect on the total range when deployed horizontally as can be seen in Table 4. The values contained in Table 4 are not directly comparable, in part as the sensors were at different water column heights, but give an indication of the range achievable.

This study has shown that the range of the single central beam on the Nortek Signature 500 is flow speed specific. In order for a horizontal Nortek Signature 500 to reach the free stream of an O${\;\;}_{2}$ turbine according to the TS 62600-200 [12], the ADCP would have to range 56.6 m under all flow conditions. This is only possible with the acceptance of 90% data loss for the Nortek Signature 500 used in this study, according to Table 3.

#### 4.1.2. Incident Flow from Divergent Beams

It is important that, within TS 62600-200, the incident flow measured represents an average of the flow speed experienced by the entire rotor diameter [12]. For a horizontal ADCP, this manifests as a beam cone the diameter of the rotor at the measurement bin. In the instance that a single ADCP beam ranges into the free stream for a specific tidal turbine, it is likely that the beam cone of a single beam will not diverge to the rotor diameter for larger turbines.

In these cases, in order to have a diverged acoustic cone the size of the rotor area, a multiple beam approach must be adopted. Figure 8 shows that beam combination is an important consideration when using a horizontally mounted ADCP in a multi-beam configuration. It is likely that one divergent beam will strike a boundary before the location of the free stream: either the sea surface (as seen in Figure 6) or the sea floor for turbines placed lower in the water column.

The transverse flow homogeneity assumed in the combination of multiple beams for instrument coordinate flow speed transformation is inherently flawed for a horizontally mounted ADCP. This is because vertical shear profiles through the water column ensure that the flow speeds across the measurement area differ. At the limit of $Z_{1,2,3,4}$, 17 m from the sensor head, the beam cone of the 25° divergent beams is 15.8 m in diameter. The difference in flow speed at the top and bottom of this beam cone can be assessed by looking at the shear profile, *U*, given by Equation (1), where $U_{ref}$ is a reference velocity, *h* is the height above the seabed, $h_{ref}$ is the height at which $U_{ref}$ occurs, and α is the shear profile exponent. Assuming a ${\frac{1}{5}}^{th}$ shear profile exponent [17] and a water depth of 35 m for the site, the flow speed difference between the top and bottom of the beam cone on the *y*-axis (see Figure 4) is as much as 14% 17 m in front of the sensor head. The shear profile has less effect on $Z_{1,3}$ as beam 1 and 3 are on the horizontal plane and so only diverge at the beam width on the y-axis (see Figure 4). In this case, only a 2% difference in flow speed between the upper and lower cone limits would be expected at 2D (56.6 m) in front of the sensor head. That said, $Z_{1,2,3,4}$ has diverged to ±8 m on the *x*-axis and ±8 m on the *y*-axis (see Figure 4) by 17 m, while $Z_{1,3}$ has diverged ±26 m on the *x*-axis and only ±1.4 m on the *y*-axis by 56.6 m. In both cases, the beam cone is not the same size and shape at the measurement location as the turbine rotor.

(1) $$U\left(h\right)=U_{ref}{\left(\frac{h}{h_{ref}}\right)}^{\alpha }\mathrm{for}\;h\geq 0$$

Figure 8 shows that the single-beam, Beam 5, measure of *Z* velocity ($Z_{5}$) is consistently lower than the transformed divergent beam recording in both the 4-beam and 2-beam case ($Z_{1,2,3,4}$ and $Z_{1,3}$). A power curve created using Beam 5 would, therefore, be more optimistic than a power curve using either of the divergent beam combinations presented.

### 4.2. Additional Applications of a Horizontal Hub Height ADCP

While a hub-mounted ADCP has been shown to provide a practical solution to floating tidal energy inflow measurement, the sensors setup can be leveraged for advancements in understanding in other areas. The O2 does not vane between tides, resulting in the horizontal ADCP measuring the inflow on one tide and the wake on the next. Profiling along the axis of wake propagation enables a time domain view from a single profile. How this profile changes with incident flow speed, and between operating states, can also be analysed for array configurations and downstream turbine interactions.

The live and uninterrupted recording of incident flow towards a turbine from a horizontal hub height ADCP presents the possibility for controller applications. Feed forward turbine control, like that already used in the wind industry [18,19], could reduce loads on a turbine, enabling a further reduction in costs for the developer.

## 5. Conclusions

The application of turbine-based sensors is key for floating tidal energy as it avoids the need for additional seabed operations. Incident flow measurement is, however, a difficult parameter to record from onboard a turbine due to the necessity for measurements to be outwith the influence of the turbine itself. Horizontal ADCPs represent a realistic means of turbine incident flow measurement.

This study shows that compliance with IEC-prescribed free stream range requirements for large tidal turbines is a challenge for horizontal ADCPs. Large turbines require a greater horizontal ADCP range as ’free-stream’ location is a function of turbine diameter. As a result, more data are lost from a horizontal ADCP at the free stream of a larger tidal turbine. This has implications for uncertainty calculations. These findings pertain to the Nortek Signature 500 used in this study. Future work could apply the methodology contained in this paper to other ADCPs in order to determine a comparable metric as to how other sensors are affected by a horizontal deployment.

Standard compliant incident flow measurement for a tidal turbine power performance assessment requires flow averaged over the entire rotor diameter. This study shows that the use of divergent beams to obtain a ’rotor area-sized beam cone’ can have implications on accuracy on flow speed measurements. The choice of beams is also important for divergent beam cone range.

Overall, while the deployment of horizontal turbine-mounted ADCPs offer a lower operational cost option for incident flow measurement as well as a number of additional potential use-cases, ADCP configuration and setup are important considerations prior to deployment.

## Figures and Tables

**Table 1:** Beam filters applied to horizontal ADCP data set.

Test ID	Test Function
T6	Flag bin if amplitude is less than 30 dB
T8	Flag bin if correlation is less than 80%
T15	Flag all bins if pitch, heading, or roll rate is more than 2.5°/s
T18	Flag if amplitude is not within 0.1 dB of previous bin
JDK1	Flag if velocity in bin is not within ±0.5 m/s of the median of beam for that time

**Table 2:** Transformed filters.

Test ID	Test Function
T10	Flag bin if velocity is greater than 8 m/s
T20	Flag bin if magnitude of velocity is more than ±0.8 m/s of the previous bin

**Table 3:** Horizontal ADCP range in meters for a given acceptable data loss percentage and flow speed.

	Data Loss				
**U (m/s)**	**90%**	**80%**	**70%**	**60%**	**50%**
−0.25	57.9	51.9	49.1	46.8	44.5
−0.75	62.6	52.2	48.5	46.0	43.7
−1.25	68.0	54.3	49.9	46.5	43.7
−1.75	68.0	58.9	52.9	49.5	46.3
−2.25	68.0	68.0	60.6	54.8	51.5
−2.75	68.0	68.0	67.2	60.0	55.4
−3.25	68.0	68.0	68.0	65.7	61.4
−3.75	68.0	68.0	68.0	68.0	67.2

**Table 4:** Range of three horizontal ADCPs deployed on full-scale tidal turbines along with manufacturer-stated range including the Nortek Signature 500 studied in this paper [8,9,16].

Sensor	Manufacturer Stated Range (m)	Horizontal Range in an Energetic Tidal Environment (m)
1 MHz Nortek Aquadopp	25	20.4
1 MHz Nortek AD2CP	-	13
Nortek Signature 500	70	31 (with 10% data loss see Figure 7).

## Data Availability

Restrictions apply to the availability of these data. Data were obtained from Orbital Marine Power and are available with the permission of Orbital Marine Power.

## Abbreviations

The following abbreviations are used in this manuscript:

ADCP	Acoustic Doppler Current Profiler
IEC	International Electrotechnical Commission
QARTOD	Quality Assurance / Quality Control of Real-Time Oceanographic Data
TS	Technical Specification
UK	United Kingdom
